# A Case of Nocturnal Enuresis, and a Case of Remittent Fever

**Published:** 1888-05

**Authors:** J. C. Holloway


### A CASE OF NOCTURNAL ENURESIS.

Miss ———, aged nineteen, dark hair and eyes, delicate, had been afflicted with nocturnal enuresis since a little girl. Some three or four "regulars" had exhausted their skill. She had been under the constant treatment of one of these for several months when I was called—September 2d, 1887. He was dismissed one day and I was called the next. Her condition was then as follows: Constant headache, consisting of sharp pains affecting both eyes, pressing outward; low spirited; *weakness of memory;* tossing about all night with moaning and talking; dim-sighted; at every attempt to read, the page becomes a blank—cannot see a letter; severe labor-like pains in small of back; feet and lower limbs swollen, cannot put on her shoes; copious emissions of urine every night—"she just floods the bed;" urine dark and has bad odor; *very hard to wake from sleep,* "I have to shake her."

Treatment.—September 2d, 1887, put her on Nux v.$^{2m}$, one dose per day for two weeks. This resulted in removing some drug symptoms, but the guiding symptoms remained the same. September 16th, when I called, the mother broke down with grief, saying her daughter was no better, and that she had given up all hope. I told her to not become discouraged, that I had not yet prescribed for her actual disorder. I then gave her *one dose* Kreos.$^{50m}$ (Dr. R. B. Johnstone's "H. S.") and Sac. lac. for one week. Eight days after reported this: "Emma is feeling somewhat better, and has not 'wet the bed' since you gave her that last medicine, until last night." Gave her one more dose of the Kreos.$^{50m}$. November 10th, reported much improved. Edematous condition of feet and limbs all gone, appetite good, sleep natural, and no "wetting the bed." Sac. lac. continued. February 10th, 1888, the girl is fresh and healthy looking, no swelling of limbs or feet, no back trouble, no headache (or but slight at times), can read as well as any one, and has never "wet the bed" since taking the second dose of Kreos.$^{50m}$.

Besides the Nux v.$^{2m}$, which she took two weeks, the two doses of Kreos.$^{50m}$ is all the medicine she has received. If the trouble is ever reported to me as having returned, I shall give her one dose of Kreos.$^{100m}$, Johnstone's "H. S." The "dose" of which I speak is a few No. 5 pellets.

### A CASE OF REMITTENT FEVER.

Young man, aged twenty-two, black eyes and hair, has been chilling four weeks. A "regular" has been giving "heroic" doses of Quinine and "some other stuff." Still, he chills every other day, beginning about ten A. M. On hearing this, I proposed to give him enough medicine to last over two chill days, at which time I would return, take his symptoms, and cure the disorder. Explained to him why this was necessary. He consented. Put one dose of Sep.$^{50m}$ on his tongue and left Sac. lac. Returned at appointed time and found following: Had had the two chills. Came on between nine and ten A. M.

PRODROME.—Flushes of heat and nausea. No thirst.

CHILL.—No thirst. Can't get warm even by a stove.

HEAT.—Great thirst. Between chill and heat much *sour* vomiting (*bitter* vomit between chill and heat, Eup. perf., Ipec.).

SWEAT.—Profuse.

Was hungry each day, but few mouthfuls filled him up. There was no red, sandy sediment in urine.

TREATMENT.—Gave him one dose Lyc.$^{43m}$. He never had another chill from that day to this. That was November 11th, '87; this is February 10th, '88. He only received the one dose (few No. 5 pellets).

I have reported these two cases in compliance with a promise made to some of my classmates who "took no stock" in Professor Kent's "private talks," because they "took no stock" in high potencies. Well, boys, as I told you then so I tell you now: They work like lightning if indicated; if not indicated, you can't give medicine strong enough to cure. I located in a community where Homœopathy was a stranger, and the foregoing are only samples of many I could report. These are good families, and those calls were my first chance at either family. They are not only now my advocates, but those two cures have made me many dollars. I keep nothing in my office lower than 200. Lower potencies might have worked in these cases, but, as you see, I did not need them. These I will leave in the market for *mongrels;* for mongrels cannot use high potencies with success. They tell us Homœopathy cannot cure intermittent fever. In each such saying they betray their own ignorance. They ought to say, "We don't know how to *cure* intermittent fever, because we don't know how to apply the homœopathic law." My potencies have been thoroughly tested in this community on intermittent fever, and I have never failed in but one case—and this fellow could not spell his own name—of course, he could not give me his symptoms. I cheerfully continued my defense against the "regulars" by keeping quiet, curing my cases and theirs, too. But please leave me my repertory and high potencies. J. C. HOLLOWAY, M. D.

